# The Unusual Late-Onset Graves' Disease following Hashimoto's Related Hypothyroidism: A Case Report and Literature Review

**DOI:** 10.1155/2020/5647273

**Published:** 2020-12-08

**Authors:** Aseel Sukik, Sara Mohamed, Mhd-Baraa Habib, Sundus Sardar, Bashar Tanous, Raad Tahtouh, Mouhand F. H. Mohamed

**Affiliations:** Internal Medicine Department, Hamad Medical Corporation, Doha, Qatar

## Abstract

*Background*. The shift of Graves' disease (GD) to Hashimoto's disease- (HD-) related hypothyroidism is well established. However, the opposite is rare. This is likely to the loss of critical thyroid mass available for stimulation by thyroid hormone receptor stimulating antibody, making this shift unusual. Herein, we report a young lady with a late shift from HD into GD and present a scoping literature review. *Case presentation*. We report a twenty-five-year-old lady with a sixteen-year-history of Hashimoto's-related hypothyroidism stable on levothyroxine. While following in the clinic, she started developing thyrotoxic symptoms in the form of anxiety, weight loss, and palpitation. Physical examination was remarkable for mild exophthalmos. The thyroid function test confirmed hyperthyroidism. Levothyroxine-induced hyperthyroidism was initially suspected; however, the symptoms did not improve despite reducing and stopping levothyroxine. Subsequent workup confirmed the diagnosis of GD. *Discussion and Conclusion*. This case highlights a unique association that has significant diagnostic and management implications. This shift should be considered when hyperthyroidism persists despite reducing or stopping levothyroxine. The diagnosis is made utilizing antibody titers and radioiodine update scan. While the management depends on the disease's stage and the treating physician preference, antithyroid agents can be used initially. Following up these patients is essential as the shift can be transient.

## 1. Introduction

Hashimoto's thyroiditis (HT) and Graves' disease (GD) are autoimmune diseases. They are the leading causes of hypothyroidism and hyperthyroidism, respectively [1, 2]. GD-related hyperthyroidism commonly converts to hypothyroidism following treatment with radioactive iodine treatment or surgery or less commonly shifts to HT-related Hypothyroidism. The latter occurs when the autoimmune response switches from thyroid stimulation to blockade [3].

In contrast, the shift from HT-related hypothyroid state to hyperthyroidism is unusual and has been rarely reported [3]. When a patient on levothyroxine for HT-related Hypothyroidism presents with thyrotoxic symptoms, levothyroxine over-replacement is usually suspected. Nonetheless, the unique possibility of transitioning to GD should be considered. We here describe a case of HT shifting to GD after sixteen years from the initial diagnosis. Additionally, we perform a scoping literature review exploring this unusual entity's mechanism, characteristics, and management.

## 2. Case presentation

Our patient is a twenty-four-year-old female with a sixteen-year history of HT-related hypothyroidism on oral levothyroxine 100 mcg once daily. She complained of occasional palpitations, nervousness, weight loss, and irregular menses. Thyrotoxicosis was confirmed with a thyroid function test, and levothyroxine dose was reduced. However, the patient continued to exhibit thyrotoxic symptoms even after completely stopping levothyroxine. On examination, the patient was calm but tachycardic and afebrile, and her blood pressure was 116/90 mmHg, weight was 58 kg, and BMI was 21.8. She was noted to have peripheral tremors. Her thyroid exam revealed the presence of diffuse nontender goiter, with no palpable nodules. Eye examination was only remarkable for mild exophthalmos, and there was no evidence of dermopathy.

**Figure 1:**
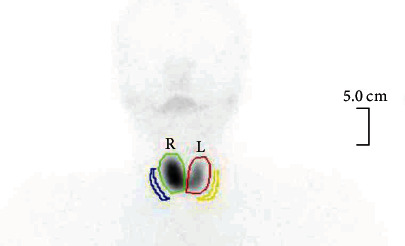
TC-99 THYROID SCINTIGRAPHY demonstrating moderate, asymmetrical enlargement of the thyroid gland, with a larger right lobe. Additionally, the right lobe demonstrates more radiotracer uptake, keeping with unilateral Grave's disease.

Thyroid function revealed depressed TSH <0.01 mIU/L (0.3–4.2) and high T4 26.4 pmol/L (11.6–21.9) (Table 1). Based on the persistence of symptoms despite stopping thyroid hormone supplementations and the presence of ophthalmopathy elements, the rare shift to GD was suspected. Autoimmune workup revealed a strong positive Anti-TSH Receptor (Anti-TSHR) titer, in addition to persistent positivity of previously positive thyroid peroxidase antibody (TPO Ab) and an increasing titer of Antithyroglobulin Ab (Table 2). Thyroid ultrasound revealed slight enlargement of the right lobe and increased vascularity in both lobes. Radioactive iodine uptake scan (RAIU) showed diffuse radiotracer uptake more prominent in the right thyroid lobe (Figure 1). Those findings kept with the shift to GD.

The patient received suppressive therapy with oral carbimazole 20 mg once daily and propranolol. A subsequent follow-up three months later showed normalization of thyroid function and improvement in clinical status.

## 3. Discussion

GD is the most common cause of hyperthyroidism [1]. It is an autoimmune disorder characterized by the presence of ophthalmopathy, dermopathy, and goiter. TSH receptor antibody is the characteristic for GD with excellent sensitivity and specificity [4]; in some cases, TPO Ab can also be detected. However, TPO Ab is usually the characteristic of HT (>90% of cases) [5]. Both GD and HT share autoimmunity pathogenesis. Although rare, these conditions can coexist [6].

Hypothyroidism follows-up to 20% of GD cases previously treated with antithyroid drugs even after [7, 8] prolonged periods [8]. Thyrotoxic manifestations in the setting of hypothyroidism usually point towards overtreatment that can be managed with modifications in thyroid hormone doses. The shift of HT-related hypothyroidism to GD is a rare entity. This is thought to be due to the critical loss of thyroid mass that can react with TSHR Ab [8]. Although rare, this shift has significant therapeutic implications and, thus, needs timely identification.

We performed a scoping review of the literature to explore this seemingly rare entity's mechanism, frequency, and characteristics. We came across a recent study by Gonzalez-Aguilera et al. [3]. On the retrospective evaluation of 2000 patients attending their clinic, they found 24 (1%) patients transforming from HD to GD. The median duration of the transformation was 18 months, majority cases were females (22/24), only four cases had ophthalmic changes, and all cases were treated initially as medication-induced hyperthyroidism. It was the persistence of symptoms (as in our case) that led to GD's workup and diagnosis. Gonzalez-Aguilera et al. postulated that this shift pathogenesis is either a shift in TSHR Ab from blocking (TSBAb) to stimulating (TSAb) or initially severe thyroid damage causing hypothyroidism that later shifts to hyperthyroidism via stimulating hormones upon thyroid gland recovery. The first explanation is more likely in our case, as she developed GD after sixteen years, which may be a long time for thyroid gland recovery.

Takasu et al. reported the second largest pool of adult patients (8 patients) with extended follow-up [9]. They suggested a classification of this cohort of patients based on the presence of TSAb and the persistence of symptoms to three groups. The first group is transient hyperthyroidism related to GD after hypothyroidism; the second group is persistent GD′-related hyperthyroidism after hypothyroidism. Finally, the third group is persistent hypothyroidism with positive TSAb. In their series, most patients (71%, *n* = 5/7) were transient, not needing specific antithyroid treatment. In two cases, the symptoms persisted, necessitating antithyroid treatment (2/7). One case had positive TSAb, however, without developing hyperthyroidism. Our literature search otherwise revealed additional individual case reports [8, 10–16]. Kamath et al. in 2012 suggested a role for the immunological shift as a possible mechanism, concluding that this theory remains only speculative at this stage [8].

The unilateral GD seen in our patient is relatively rare. It is a condition typical of Graves' symptoms, signs and supporting investigations are present, but radiotracer uptake is confined more to a single thyroid lobe [16]. Our patient had mild exophthalmos but did not have other typical Grave's orbitopathy features, usually present in almost half of GD patients [17].

Our case report is unique, given that the shift to GD occurred after sixteen years following the initial diagnosis of HD, while in other reported cases, the shift tends to occur earlier in the disease course. This indicates that this shift can occur at any time. TPO Ab remained positive, with the finding of TSHR Ab (TSHR Ab levels were not measured earlier). The patient remained well on follow-up taking antithyroid treatment. Considering the possibility of transient hyperthyroidism state, which is mostly the case in our patient, we will monitor the patient closely for the development of signs or symptoms of hypothyroidism necessitating modifications or cessation of antithyroid treatment.

## 4. Conclusions

GD should be suspected when HT patients develop thyrotoxic symptoms that do not improve after reducing or stopping levothyroxine. The diagnosis relies upon good history and physical exam followed by supportive antibody titers and radioiodine uptake scan. Our case indicated that HT's shift to GD could be late. This cohort of patients should be followed up closely as the majority may transform back to hypothyroidism.

The patient was started on levothyroxine in October 2019. Abbreviations: WBC = white blood cells, RBC = red blood cells, Hb = hemoglobin, ALT = alanine aminotransferase, AST = aspartate aminotransferase, TSH = thyroid stimulating hormone, *T*3 = Triiodothyronine, and *T*4 = thyroxine.

## Figures and Tables

**Table 1:** The patient's laboratory test results.

Test (normal range/unit)	January 2020	November 2019	September 2019	February 2019	November 2017
WBC (4–11 × 10 ^ 3/uL)		6.8	7.1	7.4	8.5
RBC (2.8–4.8 × 10 ^ 6/uL)		4.8	4.2	4.4	4.3
Hb (12–15 gm/dL)		13.8	12.4	13.1	13.1
Platelets (150–400 × 10 ^ 3/uL)		293	325	396	263
Urea (2.7–8 mmol/L)		3.5	3.8	3.4	3.6
Creatinine (44–8 umol/L)		45	48	55	64
Sodium (135–145 mmol/L)		145	143	143	141
Potassium (mmol/L 3.5–5.1)		3.3	3.8	4.7	3.7
Glucose (3.3–5.5 mmol/L)		4.6	5.5	5.8	4.4
ALT (0–33 U/L)		30	34	29	9
AST (0–33 U/L)		26	21	18	14
TSH (0.3–4.2 IU/L)	2.39	<0.01	0.01	<0.01	1.64
Free T3 (3.7–6.4 pmol/L)		7.4	8.3	10.8	
Free T4 (11.6–21.9 pmol/L)	12	23.3	26.4	37.5	19.79

**Table 2:** Autoantibodies' trend during the clinical follow-up.

Antibody	2015	2019
TPO Ab (0–34 IU/ml)	>1000	>600
Antithyroglobulin^*∗*^	0.93 (<0.6 Units)	585 (0–115 IU/mL)
Anti-TSHR (<1.75 IU/L)	___	12.2

^*∗*^Different units were used for the antithyroglobulin antibodies test. The first value was indicative of weak positivity; however, the second reading was strongly positive. TPO Ab = thyroid peroxidase antibody, Anti-TSHR = anti-thyroid-stimulating hormone receptor antibody.

## Data Availability

Further details about the case are available upon request.
